# *Helicobacter pylori* infection‐induced H3Ser10 phosphorylation in stepwise gastric carcinogenesis and its clinical implications

**DOI:** 10.1111/hel.12486

**Published:** 2018-04-15

**Authors:** Tao‐Tao Yang, Na Cao, Hai‐Hui Zhang, Jian‐Bo Wei, Xiao‐Xia Song, Dong‐Min Yi, Shuai‐Heng Chao, Li‐Da Zhang, Ling‐Fei Kong, Shuang‐Yin Han, Yu‐Xiu Yang, Song‐Ze Ding

**Affiliations:** ^1^ Department of Gastroenterology and Hepatology People's Hospital of Zhengzhou University Zhengzhou China; ^2^ Henan Police College Zhengzhou China; ^3^ Department of Gastroenterology and Hepatology Xinxiang Medical University Xinxiang China; ^4^ Department of Pathology People's Hospital of Zhengzhou University Zhengzhou China

**Keywords:** epigenetic, gastric cancer, H3 serine 10 phosphorylation, *Helicobacter pylori*, histone modification

## Abstract

**Background:**

Our previous works have demonstrated that *Helicobacter pylori* (*Hp*) infection can alter histone H3 serine 10 phosphorylation status in gastric epithelial cells. However, whether *Helicobacter pylori*‐induced histone H3 serine 10 phosphorylation participates in gastric carcinogenesis is unknown. We investigate the expression of histone H3 serine 10 phosphorylation in various stages of gastric disease and explore its clinical implication.

**Materials and Methods:**

Stomach biopsy samples from 129 patients were collected and stained with histone H3 serine 10 phosphorylation, Ki67, and *Helicobacter pylori* by immunohistochemistry staining, expressed as labeling index. They were categorized into nonatrophic gastritis, chronic atrophic gastritis, intestinal metaplasia, low‐grade intraepithelial neoplasia, high‐grade intraepithelial neoplasia, and intestinal‐type gastric cancer groups. *Helicobacter pylori* infection was determined by either ^13^C‐urea breath test or immunohistochemistry staining.

**Results:**

In *Helicobacter pylori*‐negative patients, labeling index of histone H3 serine 10 phosphorylation was gradually increased in nonatrophic gastritis, chronic atrophic gastritis, intestinal metaplasia groups, peaked at low‐grade intraepithelial neoplasia, and declined in high‐grade intraepithelial neoplasia and gastric cancer groups. In *Helicobacter pylori*‐infected patients, labeling index of histone H3 serine 10 phosphorylation followed the similar pattern as above, with increased expression over the corresponding *Helicobacter pylori*‐negative controls except in nonatrophic gastritis patient whose labeling index was decreased when compared with *Helicobacter pylori*‐negative control. Labeling index of Ki67 in *Helicobacter pylori*‐negative groups was higher in gastric cancer than chronic atrophic gastritis and low‐grade intraepithelial neoplasia groups, and higher in intestinal metaplasia group compared with chronic atrophic gastritis group. In *Helicobacter pylori*‐positive groups, Ki67 labeling index was increased stepwise from nonatrophic gastritis to gastric cancer except slightly decrease in chronic atrophic gastritis group. In addition, we noted that histone H3 serine 10 phosphorylation staining is accompanied with its location changes from gastric gland bottom expanded to whole gland as disease stage progress.

**Conclusions:**

These results indicate that stepwise gastric carcinogenesis is associated with altered histone H3 serine 10 phosphorylation, *Helicobacter pylori* infection enhances histone H3 serine 10 phosphorylation expression in these processes; it is also accompanied with histone H3 serine 10 phosphorylation location change from gland bottom staining expand to whole gland expression. The results suggest that epigenetic dysregulation may play important roles in *Helicobacter pylori*‐induced gastric cancer.

## 1 INTRODUCTION

Gastric cancer (GC) is the fifth most common malignancy and the third leading cause of cancer‐related death worldwide. Nearly half of the disease burden occurs in Eastern Asia, particularly in China, South Korea, and Japan.^1^ GC can be subdivided into intestinal type and diffuse type based on Lauren's classification.^2^ The development of noncardia intestinal‐type GC follows a well‐defined histological sequence of progression in *Helicobacter pylori* (*H. pylori, Hp*)‐infected gastric mucosa, initiating as chronic gastritis followed by atrophy, intestinal metaplasia, dysplasia, and GC.^3^, ^4^, ^5^, ^6^ *H. pylori* infection is the known single most important factor for GC, and eradication of *H. pylori* not only cures gastritis but also prevents the progression to long‐term complications, such as atrophic gastritis, intestinal metaplasia, recurrence of ulcers and reduces the incidence of GC. However, how *H. pylori* infection results in the initiation of GC remains unknown.

Histone modification plays important roles in various cellular functions and cancer; they affect gene expression by changing the state of transcription factors accessing to chromatin.^7^, ^8^, ^9^, ^10^, ^11^ The post‐translational modification of histone tails also affects different levels of DNA organization, including acetylation, phosphorylation, methylation, ubiquitylation, and ADP ribosylation on it amino acid residues,^8^, ^11^, ^12^, ^13^ the combination of these modifications are important marks of epigenetics, commonly known as “histone code.” Increasing evidences have indicated its role in tumor initiation and development.^14^

Several reports have indicated that histone H3 serine 10 (H3Ser10) is involved in epithelial carcinogenesis. For example, phosphorylation of H3Ser10 (p‐H3Ser10) is an essential regulatory mechanism for epidermal growth factor (EGF)‐induced neoplastic cell transformation, which involves the induction of *c‐fos* and *c‐jun* promoter activity.^15^, ^16^ Overexpression of phosphorylated histone H3 in gastric tissue has been reported as an indicator of poor prognosis in gastric adenocarcinoma patients.^17^ Increased p‐H3Ser10 is critical in EB virus‐induced carcinogenesis of nasopharyngeal carcinoma;^18^ active protein‐1, mitogen‐ and stress‐activated kinase 1 (MSK1) kinase activity and phosphorylation also participate in this process.^18^ Our previous work has demonstrated that *H. pylori* infection alters histone modification and host response via *cag*PAI‐dependent mechanisms; we showed that wild‐type *H. pylori* induced time‐ and dose‐dependent dephosphorylation of H3Ser10 and decreased acetylation of H3 lysine 23 (H3K23ac), but have no effects on seven other specific modifications.^19^ However, whether *H. pylori* infection affects histone modifications in human stomach and its role in gastric carcinogenesis remains elusive.

In this report, we evaluate the expression pattern of p‐H3Ser10 in stomach biopsy samples in chronic gastric disease and patients with GC, and assess its role in the stepwise gastric disease progress. The results indicate a strong correlation of histone H3Ser10 phosphorylation with chronic gastric disease progression and implicate its role in *H. pylori‐*induced gastric carcinogenesis.

## 2 MATERIALS AND METHODS

### 2.1 Patients

From November 2015 to May 2017, 134 subjects were enrolled in this study at People's Hospital of Zhengzhou University, China, including 83 males and 51 females, mostly due to upper gastrointestinal tract discomfort. Inclusion criteria include no proton‐pump inhibitor use within past 2 weeks; no antibiotic and bismuth compound use over the past 4 weeks and no previous treatment. During the histological section re‐evaluation process, two patients (2 males in *H. pylori*‐positive) were excluded due to their original diagnosis could not be confirmed; three other patients were excluded as they were later confirmed as mixed‐type GC in *H. pylori*‐negative intestinal‐type GC group. Overall, the survival data were available in 129 of 134 cases (96.27%).

[Correction added on 25 April 2018, after first online publication: The number of subjects enrolled were updated to 83 males and 51 females in this version.]

^13^C‐urea breath test (^13^C‐UBT) was performed before and after patient underwent gastroscope examination. All biopsy samples were taken from gastric antrum and noncardia regions. They were categorized into nonatrophic gastritis (NAG), chronic atrophic gastritis (CAG), intestinal metaplasia (IM), low‐grade intraepithelial neoplasia (LGIN), high‐grade intraepithelial neoplasia (HGIN), and intestinal‐type GC groups. Diagnosis of *H. pylori* infection was made based on the positive result of either ^13^C‐UBT or immunohistochemistry staining of *H. pylori*. All participating patients signed informed consent; the research protocol was approved by the Ethics Committee of People's Hospital of Zhengzhou University.

### 2.2 Reagents and immunohistochemistry staining

Polyclonal rabbit antihistone H3 phospho‐specific at serine 10 antibody was purchased from Cell Signaling Technology (Cell Signaling Technology, Cambridge, MA, USA, #9701, dilution 1:800). Monoclonal mouse anti‐human Ki‐67 (cloning number: MIB‐1) and anti‐*H. pylori* antibodies (cloning number: MX014) were purchased from MXB Biotechnologies (MXB Biotechnologies, Fuzhou, China), and the antibodies were diluted in antibody diluent (product number: ABD‐0030, MXB Biotechnologies, Fuzhou, China).

Immunohistochemistry staining (IHS) was performed on 4‐μm‐thick serial sections derived from formaldehyde‐fixed paraffin blocks. Diagnoses were made with one slide stained with hematoxylin and eosin (HE). Histological re‐examination of primary tissue sections was carried out for all cases to confirm the diagnosis. IHS was performed with LEICA BOND‐MAX automated slide staining system, using the Bond Polymer Refine Detection (Leica Biosystems Newcastle Ltd, Newcastle, United Kingdom) and aforementioned antibodies. Three serial sections for immunohistochemistry were stained with *H. pylori*, p‐H3Ser10, and Ki67 antibodies, respectively. Olympus microscopes (BX43, Olympus, Tokyo, Japan) were used to observe the section and take photographs for staining evaluation.

### 2.3 Evaluation of immunostaining

Immunostaining results were scored as described previously.^20^, ^21^, ^22^ All sections were evaluated blindly by two independent reviewers (Song XX and Yang TT) with high degree of concordance. Both p‐H3Ser10 and Ki67 showed nuclear expression. The sections were manually evaluated, and staining cells were counted under the light microscope (10 × 20). Percentage of positive cells in the section was graded as follows: 0, none; 1, ≤10%; 2, 11%‐50%; 3, 51%‐80%; and 4, >80%. Intensity of immunostaining was rated as follows: 0, none; 1, light brown; 2, brown; and 3, dark brown. Each section was scored in 3‐8 fields depending on the scope of lesions. Labeling index (LI) was calculated by multiplying percentage positive cells rating by intensity rating in every field; these results were averaged as the LI of each section. The LI of each patient within the group was averaged to generate mean value and standard deviation; they were compared among different groups.

In addition, the localization of p‐H3Ser10‐ and Ki67‐immunopositive cells in gastric gland in different disease groups was investigated, they were visualized and recorded under microscope as mentioned above, and the percentage of stained cells in each location of gastric gland in every section were then averaged to generate a mean value and represented the percentage of p‐H3Ser10‐ or Ki67‐positive cells' locations in each gastric disease group. *H. pylori* infection status was categorized as *H. pylori*‐positive and *H. pylori*‐negative.

### 2.4 Statistical analysis

The data were analyzed using SPSS for Windows (Version 22, IBM Corp., New York, NY, USA). All data were tested for normal distribution by Kolmogorov‐Smirnov test and for homogeneity of variances by Levene's test. Data of normal distribution and similar variances were tested by Student's “*t*” test for two independent samples comparison, and ANOVA was used for multiple comparisons among different groups and all data expressed as mean ± standard deviation (SD). Comparison of ratios between different groups was made by chi‐square test. The correlations of LI among different groups were assessed using Spearman's correlation coefficient test. *P *<* *.05 was considered statistically significant, which was derived from two‐tailed tests. The variance was of highly statistically significant when *P *<* *.01.

## 3 RESULTS

### 3.1 Correlation of patient clinical data with *H. pylori* infection status

A total of 129 patients were analyzed in this study; their average age is 59 (range from 13 to 82) years. The clinicopathological characteristics are summarized in Table 1. The patients were categorized into *H. pylori*‐infected and noninfected groups; the baseline level of clinical features such as age, gender, disease stage, and histology classifications showed no statistical difference between the two groups (*P* > .05, Table 1).

**Table 1:** Clinicopathological patient characteristics and correlation with the *Helicobacter pylori* infection status

	Total	*Hp*(+)	*Hp*(−)	Value *P*
Age(years)	59.13 ± 1.09	58.90 ± 11.55	59.33 ± 13.13	.85^a^
Gender				
Male	78	37	41	.63^a^
Female	51	22	29	
Histology classification				
NAG	25	10	15	.18^a^
CAG	20	11	9	
IM	29	9	20	
LGIN	21	14	7	
HGIN	12	6	6	
GC(Intestinal‐type)	22	9	13	

NAG, nonatrophic gastritis; CAG, chronic atrophic gastritis; IM, intestinal metaplasia; LGIN, low‐grade intraepithelial neoplasia; HGIN, high‐grade intraepithelial neoplasia; GC, gastric cancer.

Student's “*t*” test.

a Chi‐square test.

### 3.2 Labeling index of p‐H3Ser10 and Ki‐67 in *H. pylori*‐infected and noninfected groups

**Figure 1:**
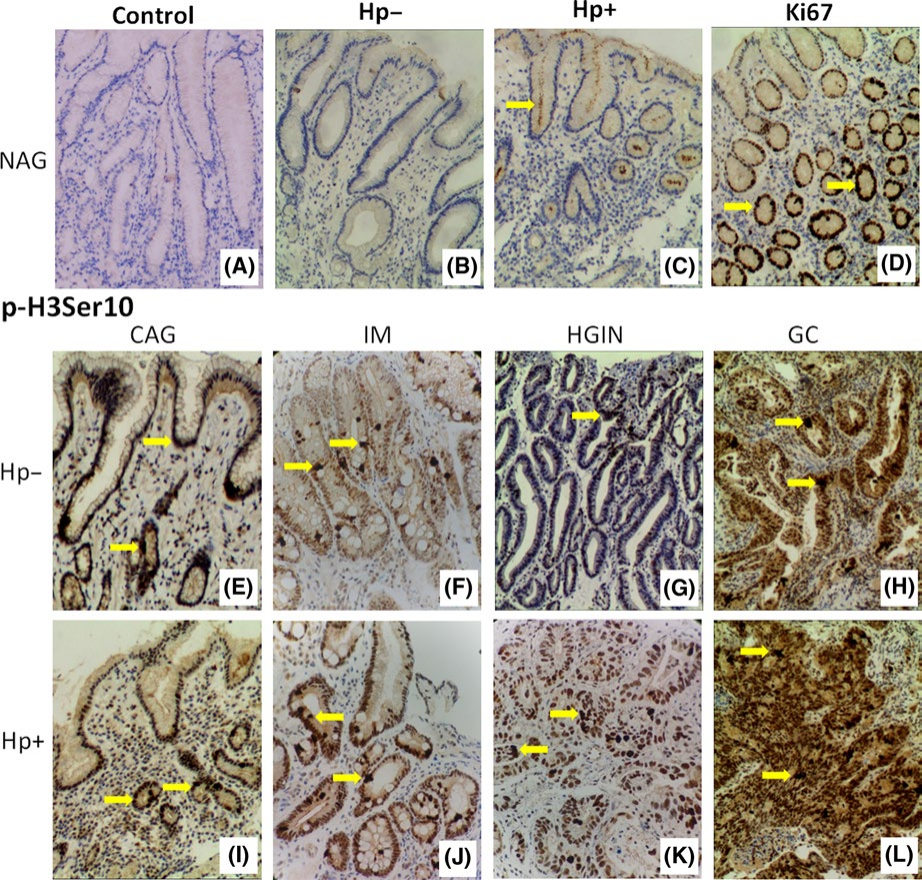
Representative photographs of p‐H3Ser10 and Ki67 immunostaining in gastric tissues. A, Negative control in nonatrophic gastritis (NAG) patients in which primary antibody has been omitted (control); B, *Helicobacter pylori*‐negative (*Hp*−) in NAG patient; C, *H. pylori*‐positive (*Hp*+) in NAG patient; D, Ki67 in *H. pylori*‐positive NAG patient; E‐L, p‐H3Ser10 staining, E and I, chronic atrophic gastritis (CAG); F and J, intestinal metaplasia (IM); G and K, high‐grade intraepithelial neoplasia (HGIN); H and L, intestinal‐type gastric cancer (GC). H3Ser10‐stained cells are located mainly in the bottom and body of gastric gland in NAG (E and I). Almost all cells show nuclear immunoreactivity in IM, GC, and *H. pylori*‐positive HGIN (F, H, J, K, and L). Arrows indicate representative immunostaining cells. Original magnifications ×100 (A‐L)

A total of 59 patients were grouped into *H. pylori‐*positive and 70 patients in *H. pylori*‐negative groups; total LI of p‐H3Ser10 and Ki‐67 was compared between the two groups. We noted that *H. pylori* infection increased overall LI of p‐H3Ser10 significantly when compared with *H. pylori*‐negative group (6.31 ± 2.22 vs 5.35 ± 2.10, *P *<* *.05); there was no difference on the overall level of Ki‐67 expression between two groups (*P *>* *.05) (Table 2). However, *H. pylori* induced higher Ki‐67 expression level in intestinal‐type GC over noninfected GC controls (*P *<* *.01, Table 3). Representative IHS images of p‐H3Ser10 in different groups are presented in Figure 1. Both Ki67 and H3Ser10 phosphorylation showed mild to strong nuclear expression.

**Table 2:** H3Ser10 phosphorylation and Ki67 expression in *Hp*(+) and *Hp*(−) groups

	*Hp*(+)	*Hp*(−)	Value *P* b
H3Ser10 phosphorylation	6.31 ± 2.22	5.35 ± 2.10	.013^a^
Ki67	7.31 ± 2.15	7.22 ± 1.63	.799

a Value that is statistically significant (<.05).

b Student's “*t*” test.

**Table 3:** H3Ser10 phosphorylation and Ki67 expression between each group of *Hp*(+) vs *Hp*(−)

Groups	H3Ser10 phosphorylation			Ki67		
	*Hp*(+)	*Hp*(−)	Value *P* a	*Hp*(+)	*Hp*(−)	Value *P* a
NAG	3.52 ± 1.39	5.24 ± 1.98	.018*	6.74 ± 1.89	6.92 ± 0.98	.746
CAG	5.58 ± 1.21	4.32 ± 1.40	.044*	5.82 ± 1.32	5.82 ± 1.50	.991
IM	6.39 ± 1.07	5.79 ± 2.12	.325	6.71 ± 1.93	7.79 ± 1.06	.060
LGIN	8.46 ± 1.94	7.25 ± 2.06	.205	6.85 ± 1.42	6.48 ± 1.35	.568
HGIN	7.50 ± 1.12	3.08 ± 1.06	.000**	8.00 ± 1.40	7.20 ± 2.62	.523
GC(Intestinal‐type)	6.09 ± 2.04	5.53 ± 1.96	.527	10.59 ± 1.61	8.05 ± 1.97	.005**

NAG, nonatrophic gastritis; CAG, chronic atrophic gastritis; IM, intestinal metaplasia; LGIN, low‐grade intraepithelial neoplasia; HGIN, high‐grade intraepithelial neoplasia; GC, Gastric cancer.

The asterisks indicate values that are statistically significant (<.05).

a Student's “*t*” test.

### 3.3 Labeling index of p‐H3Ser10 and Ki‐67 among different stages of gastric disease

**Figure 2:**
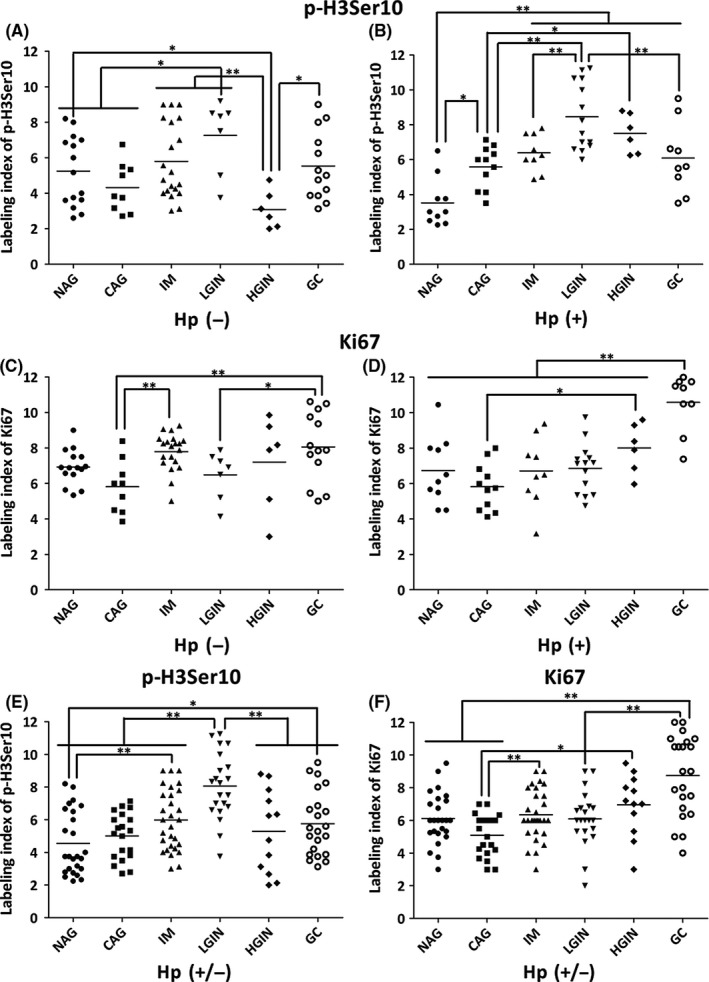
Labeling index of p‐H3Ser10 and Ki67 in various stages of gastric disease. Labeling index (LI) of p‐H3Ser10 (A and B and E)‐ and Ki67 (C and D and F)‐stained cells was generated by visualizing and recording the image data under microscope; it was calculated as mentioned in method and expressed as mean ± SD in each group. The average level of p‐H3Ser10 and Ki67 LI among various stages of gastric disease was compared by ANOVA. X‐axes indicate different stages of disease; Y‐axes indicate LI of p‐H3Ser10 and Ki67. Dots represent LI of either p‐H3Ser10 or Ki67 in each group; the short bars between dots indicate mean value of each group. One asterisk represents value that is statistically significant (*P *<* *.05); two asterisks indicate value that is highly statistically significant (*P *<* *.01). Nonatrophic gastritis, NAG; chronic atrophic gastritis, CAG; intestinal metaplasia, IM; low‐grade intraepithelial neoplasia, LGIN; high‐grade intraepithelial neoplasia, HGIN; intestinal‐type gastric cancer, GC;* Helicobacter pylori* infection status, *H. pylori*‐positive; *H. pylori* noninfection status, *H. pylori*‐negative; *H. pylori* infection or noninfection status, *H. pylori* (+/−)

In *H. pylori*‐negative patient groups, LI of p‐H3Ser10 was increased as disease progress from NAG to CAG, IM, and LGIN groups, and peaked at LGIN; its level declined in HGIN and GC groups (Figure 2A). In *H. pylori*‐infected groups, LI of p‐H3Ser10 followed the similar pattern as above; however, increased LI of p‐H3Ser10 was noted in all groups when compared with *H. pylori*‐negative groups, except in NAG group, whose expression level was lower when compared with *H. pylori*‐negative NAG group (*P *<* *.05, Figure 2B and Table 3).

The general LI of Ki67 showed a trend of gradually increasing from NAG stage to GC stage groups, except slightly decreased in CAG groups. In *H. pylori‐*negative groups, the LI of Ki67 was significantly higher in GC than that in CAG and LGIN groups (*P *<* *.05); and in IM group compared with CAG group (7.79 ± 1.06 vs 5.82 ± 1.50, *P *<* *.01) (Figure 2C and Table 3). In *H. pylori*‐infected patients, Ki67 LI followed the similar pattern as in *H. pylori‐*negative groups and peaked at GC groups (*P *<* *.01); higher level of Ki67 LI in HGIN was noticed when compared with CAG group (*P *<* *.05) (Figure 2D and Table 3).

Irrespective of *H. pylori* infection status, the overall staining level of p‐H3Ser10 and Ki‐67 in *H. pylori*‐positive and ‐negative polled groups showed similar pattern as *H*. *pylori*‐infected patient group as disease progress from NAG to GC stages of disease (Figure 2E and 2F).

To explore the correlation between p‐H3Ser10 and Ki‐67 among all these disease groups, Spearman's correlation coefficient was used to assess their relationship; the results indicated that there was no significant correlation between p‐H3Ser10 and Ki‐67 LI among different disease groups (*P *>* *.05).

### 3.4 Location changes in p‐H3Ser10‐ and Ki‐67‐stained cells among various stages of gastric disease

As location of p‐H3Ser10‐ and Ki‐67‐stained cell in gastric gland may be closely linked to its function, and their distribution in stepwise gastric carcinogenesis has not been explored, we therefore investigated their location changes during disease progress. The results indicated that locations of p‐H3Ser10‐stained cells were gradually moved from bottom of gastric gland to the whole gland distribution as disease progress from benign stage to GC.

**Figure 3:**
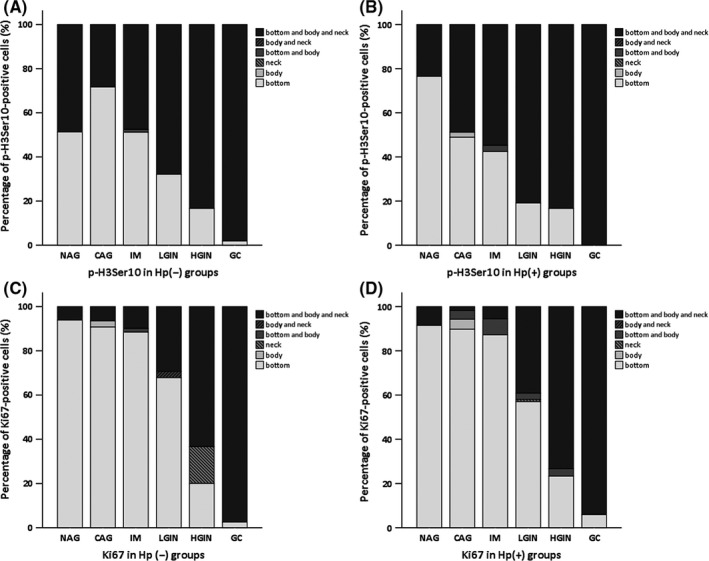
Location changes in p‐H3Ser10‐ and Ki67‐stained cells in stepwise gastric disease. Location of p‐H3Ser10 (A and B)‐ and Ki67 (C and D)‐positive cells in gastric gland was visualized and recorded under microscope; the percentage of stained cell in each location of gastric gland in every section was evaluated to generate mean value. X‐axes indicate different stages of disease; Y‐axes indicate the percentages of p‐H3Ser10‐ or Ki67‐positive cell's location. Nonatrophic gastritis, NAG; chronic atrophic gastritis, CAG; intestinal metaplasia, IM; low‐grade intraepithelial neoplasia, LGIN; high‐grade intraepithelial neoplasia, HGIN; intestinal‐type gastric cancer, GC;* Helicobacter pylori* infection status, *H. pylori*‐positive; *H. pylori* noninfection status, *H. pylori*‐negative

In *H. pylori‐*negative groups, approximately half of the p‐H3Ser10‐stained cells located at bottom of gastric gland in NAG, CAG, and IM groups, and they start to spread to the body, neck, and whole gland as disease progress from LGIN to GC stage of disease (67.86% to 98.08%) (Figure 3A). In *H. pylori*‐positive groups, similar distribution pattern of p‐H3Ser10‐stained cells was noticed as in *H. pylori‐*negative groups, but staining level of p‐H3Ser10‐positive cells located in the bottom of gastric gland was higher in NAG group than that in *H. pylori‐*negative NAG controls (76.5% vs 51.33%, Figure 3B).

For Ki67, the positive stained cells mainly lie in the bottom of benign gastric gland in NAG, CAG, and IM groups; as disease progress, they start to become whole gland distribution (Figure 3C and D). There were no differences in the percentages of Ki67‐stained cells in each location between *H. pylori*‐infected and noninfected groups (Figure 3C and D).

## 4 DISCUSSION

Recent progresses have demonstrated important roles of post‐translational histone modifications in various biology processes and cancer. However, relatively few studies have evaluated their impact in gastric carcinogenesis, especially during *H. pylori* infection, a critical factor that causing chronic gastritis and gastric cancer. In this work, the LI of p‐H3Ser10 and Ki67 in six types of gastric lesions during multistep carcinogenesis in the presence or absence of *H. pylori* infection was analyzed. We noted significant links between phosphorylated H3Ser10 staining and various stages of gastric disease.

Epigenetic dysregulation is being increasingly recognized as a hallmark of cancer. Accumulating studies have begun to investigate histone modifications in the pathogenesis of cancer development and progression. Histone modifications play critical roles in chromatin regulation, gene transcription, and nuclear architecture, thereby affect various cellular functions and participate in carcinogenesis. Phosphorylation of histone H3 has been tightly correlated with chromosome condensation during mitosis, and recently, its role in gastric carcinogenesis has been reported.^17^, ^23^, ^24^ Takahashi et al^17^ have assessed p‐H3Ser10 expression and its relation to gastric adenocarcinoma progression, in patient with increased histone H3 phosphorylation, the prognosis is worse than those with low levels of expression. Further studies have demonstrated that increased level of p‐H3Ser10 in tumor tissues is not due to changes in cell cycle but rather an interphase‐associated phenomenon, and p38‐MAPK/MSK1‐regulated increase of p‐H3Ser10 in GC is predictive of a more aggressive phenotype; this also could help defining the true negative margin of surgical resection.^24^ However, relatively few studies have investigated the role of *H. pylori* infection in these processes.

Multiple factors have been shown to affect H3Ser10 phosphorylation in epithelial cells, such as gram‐negative bacteria infection,^9^ EB virus infection,^18^ cigarette smoking,^25^ ionization radiation,^26^ chemical formaldehyde,^27^ heavy metals such as arsenite and nickel compound exposure.^28^, ^29^ p‐H3Ser10 is essential to regulate downstream gene expression such as *FOS*,* EGR1,* and *CXCL8* mRNA in cultured human cell lines and affect cellular function.^28^ However, its regulation by *H. pylori* infection in human stomach has not been evaluated.

Our previous in vitro work has demonstrated that wild‐type *H. pylori* induced time‐ and dose‐dependent dephosphorylation of H3Ser10 and decreased H3K23ac in gastric epithelial cells, which is *cag*PAI‐dependent.^19^ Several commonly expressed genes, including *CXCL8*,* PTGS2*,* CXCL2*,* PRKDC*,* DUSP4*, and *CCND1,* are not associated with or are independent of dephosphorylation of H3Ser10. However, altered *JUN* and *HSP70* gene expression is associated with H3Ser10 dephosphorylation.^19^ The present work has enriched the previously founding and noted the impact of *H. pylori* infection on p‐H3Ser10 expression at various stages of gastric carcinogenesis. Unlike in cell line studies, the present work has noted increased global p‐H3Ser10 expression in *H. pylori‐*infected stomach. The explanation for this discrepancy may lay in the fact that *H. pylori* infection in gastric tissues activates multiple cellular signal pathways in various cell types, and adding many layers of cell stimulation, which is different from single cell line exposure experiment.

The relationship of *cag*PAI status with p‐H3Ser10 in the current investigation is not clear. We have later analyzed a portion of patients for their *cag*PAI status using serum antibody, and noted that among 59 *H. pylori*‐positive patients, 34 cases (57.63%) are *cag*PAI‐positive, three cases are *cag*PAI‐negative, but in another 22 *H. pylori*‐positive cases, their *cag*PAI status cannot be determined due to lacking of material; therefore, future work is required to determine the role of *cag*PAI in H3Ser10 phosphorylation in human stomach to understand its pathogenesis.

In addition to regulating gene transcription, H3Ser10 is also a cell cycle mitotic marker in proliferating cells, and *H. pylori* has been shown to affect cell cycle progression, as several papers have indicated that *H. pylori*‐induced cell cycle arrest is independent of *cag*PAI, and both VacA and CagA also affect the cell cycle progression.^30^, ^31^ To evaluate the cell proliferation status during *H. pylori* infection in stepwise gastric carcinogenesis, we use Ki‐67 as a proliferation marker to monitor the effects of *H. pylori* on the cell proliferation in gastric tissues. The results indicate that *H. pylori* infection enhances Ki‐67 expression only in patients with GC, and there is no significant effect on overall cell proliferation; this is also in line with a few previously report with similar findings,^32^, ^33^ indicating that Ki‐67 proliferation index is a useful marker to differentiate benign and malignant lesions in gastric biopsies rather than a prognostic biomarker for patient with GC.^17^, ^34^ Moreover, there appears to be no strict linkage to cancer progression.

The location and its clinical significance of p‐H3Ser10‐positive staining cells in gastric gland have not been reported previously; the bottom and neck regions are usually where the gastric gland stem cells located, and *H. pylori* infection has recently been shown to induce expansion of this type of cells.^35^ However, the relationship of p‐H3Ser10 with gastric stem cells and its role in regulating this type of cells have yet to be established; we noted p‐H3Ser10‐staining cells were gradually expanded from the bottom of gastric gland to the whole gastric gland distribution as disease progress. The results show that *H. pylori* infection enhanced the percentage of p‐H3Ser10‐stained cells in the bottom region in NAG group when compared with *H. pylori*‐negative group; however, this difference disappeared at later stages in CAG, IM, LGIN, HGIN, and GC stages. The reason is not clear at present, probably because our case number in the study is not large enough, as strong staining throughout the gland at later stage of disease is noted, this probably indicates its link with carcinogenesis; future studies are required to understand its pathogenesis.

In summary, the current works indicate that stepwise gastric carcinogenesis is associated with altered H3Ser10 phosphorylation, *H. pylori* infection modified this process and resulted in early stage p‐H3Ser10 reduction but later stage increased expression; this is also accompanied with p‐H3Ser10 location change from bottom of gland staining expand to the whole gland expression as disease progress. The results suggest that p‐H3Ser10 may play an important role during neoplastic transformation processes; further investigation would be helpful to understand how *H. pylori* induce gastric cancer through epigenetic dysregulation.

## ETHICAL APPROVAL

This study was approved by Ethics Committee of People's Hospital of Zhengzhou University, Zhengzhou, China.

## DISCLOSURE OF INTERESTS

All the authors declare that they have no conflict of interests.

## AUTHOR CONTRIBUTION

Ding SZ, Yang YX, Han SY, and Kong LF designed the research; Yang TT, Cao N, Zhang HH, Wei JB, Yi DM, Chao SH, and Zhang LD collected the clinical data and performed the experiment; Yang TT and Song XX evaluated immunohistochemical staining; Yang TT analyzed the data; Yang TT and Ding SZ wrote the paper; Ding SZ revised the article; all authors have approved the final version of manuscript.
